# Priority Given to Technology in Government-Based Mental Health and Addictions Vision and Strategy Documents: Systematic Policy Review

**DOI:** 10.2196/25547

**Published:** 2021-05-05

**Authors:** Shalini Lal, Lyna Siafa, Hajin Lee, Carol E Adair

**Affiliations:** 1 School of Rehabilitation Faculty of Medicine University of Montréal Montréal, QC Canada; 2 Youth Mental Health and Technology Lab Health Innovation and Evaluation Hub University of Montréal Hospital Research Centre Montréal, QC Canada; 3 Prevention and Early Intervention Program for Psychosis and ACCESS Open Minds Douglas Mental Health University Institute Montréal, QC Canada; 4 Department of Medicine McGill University Montréal, QC Canada; 5 Department of Psychiatry Cumming School of Medicine University of Calgary Calgary, AB Canada; 6 Department of Community Health Sciences Cumming School of Medicine University of Calgary Calgary, AB Canada

**Keywords:** e-mental health, digital mental health, virtual care, technology, mental health, addictions, review, policy, e-health, mHealth, digital health, tele

## Abstract

**Background:**

The use of information and communication technologies (ICTs) to deliver mental health and addictions (MHA) services is a global priority, especially considering the urgent shift towards virtual delivery of care in response to the COVID-19 pandemic. It is important to monitor the evolving role of technology in MHA services. Given that MHA policy documents represent the highest level of priorities for a government’s vision and strategy for mental health care, one starting point is to measure the frequency with which technology is mentioned and the terms used to describe its use in MHA policy documents (before, during, and after COVID-19). Yet, to our knowledge, no such review of the extent to which ICTs are referred to in Canadian MHA policy documents exists to date.

**Objective:**

The objective of this systematic policy review was to examine the extent to which technology is addressed in Canadian government–based MHA policy documents prior to the COVID-19 pandemic to establish a baseline for documenting change.

**Methods:**

We reviewed 22 government-based MHA policy documents, published between 2011 and 2019 by 13 Canadian provinces and territories. We conducted content analysis to synthesize the policy priorities addressed in these documents into key themes, and then systematically searched for and tabulated the use of 39 technology-related keywords (in English and French) to describe and compare jurisdictions.

**Results:**

Technology was addressed in every document, however, to a varying degree. Of the 39 searched keywords, we identified 22 categories of keywords pertaining to the use of technology to deliver MHA services and information. The 6 most common categories were *tele* (n=16/22), *phone* (n=12/22), *tech* (n=11/22), *online* (n=10/22), *line* (n=10/22), and *web* (n=10/22), with n being the number of policy documents in which the category was mentioned out of 22 documents. The use of terms referring to advanced technologies, such as *virtual* (n=6/22) and *app* (n= 4/22), were less frequent. Additionally, policy documents from some provinces and territories (eg, Alberta and Newfoundland and Labrador) mentioned a diverse range of ICTs, whereas others described only 1 form of ICT.

**Conclusions:**

This review indicates that technology has been given limited strategic attention in Canadian MHA policy. Policy makers may have limited knowledge on the evidence and potential of using technology in this field, highlighting the value for knowledge translation and collaborative initiatives among policy makers and researchers. The development of a pan-Canadian framework for action addressing the integration and coordination of technology in mental health services can also guide initiatives in this field. Our findings provide a prepandemic baseline and replicable methods to monitor how the use of technology-supported services and innovations emerge relative to other priorities in MHA policy during and after the COVID-19 pandemic.

## Introduction

According to a Canadian survey conducted in 2018 [1], approximately 5.3 million individuals aged 12 years and older reported a need for mental health care in the previous year, yet the mental health needs of almost half of these individuals were unmet. The need to improve access and quality of mental health and addictions (MHA) services is increasingly reflected in Canadian mental health policy and practice [2]. Moreover, consequences of COVID-19 have compounded the need for mental health services [3,4], with concurrent public health guidelines for social distancing creating additional challenges to the delivery of care. These challenges have driven rapid integration of technology into the MHA system [5-8].

The use of the internet and related information and communication technologies (ICTs) to deliver or enhance mental health services (also referred to as digital mental health/digital health, e-mental health/e-health, telehealth, or virtual care) is well recognized for its potential in helping to overcome some of the barriers that individuals face in accessing mental health care [9-12]. For example, compared to conventional approaches, the use of ICTs can provide more accessible, empowering, and sustainable care [11-14]; improve access to specialist mental health care; and reduce wait times [15]. This is particularly important for rural and remote areas that have inequitable access to mental health services due to geographic distance and scarcity of resources, and for populations that are less inclined to seek help in person due to stigma [11,12]. Moreover, opportunities to receive care via ICTs also have the potential to provide individuals choice and control over their care [16].

A wide range of ICTs (eg, telephone and videoconferencing services, websites, smartphone apps, social media) have been used to develop innovations that deliver mental health services [11,12]. In general, these innovations serve the purpose of providing information and self-management tools; conducting screening and assessment; facilitating monitoring of symptoms, activities, and behaviors; and delivering psychological and social interventions (eg, peer support, counseling, case management) [11,12]. Examples include telephone-based crisis interventions [17], psychiatric assessment and treatment via videoconferencing (also known as telepsychiatry) [18,19], web-based therapeutic interventions [20,21], behavioral and psychosocial treatments using smartphone software apps and secured websites [22,23], use of sensors for patient monitoring [24], online peer support through social media [25], and virtual reality therapies [26].

In terms of evidence, there is increasing research indicating the efficacy of using ICTs to deliver mental health services and its effectiveness [20,27,28]. For example, a recent review and meta-analysis demonstrated the efficacy of using ICT-based interventions to address eating disorders under controlled trials. Specifically, digital interventions were shown to be more effective than control conditions in reducing risk factors and symptoms in prevention-focused trials and treatment-focused trials [28]. However, other types of individual-level research, including economic analyses, are still needed, in addition to broader analyses, such as studying change at the policy level.

In Canada, innovation, research, and practice on the use of ICTs in mental health care has significantly advanced over the past decade. Several examples of such advancements are provided in the gray and peer-reviewed literature [10,11], including but not exclusive to evidence-based technology-supported initiatives from the Strongest Families Institute (a not-for-profit organization originating from clinical trials conducted at the IWK Health Centre (formerly Izaak Walton Killam Health Centre) in Nova Scotia and onlinetherapyuser.ca (based on clinical research conducted at the University of Regina with eventual sustained funding received from the Saskatchewan Ministry of Health). However, the use of ICTs in mental health care is still a relatively new paradigm [11,29,30], with implementation remaining limited and fragmented [11,29] even within the context of the COVID-19 pandemic. This indicates a systemic gap between evidence and its implementation, which may in part be due to limited guidance on how to translate evidence into policy and competing priorities in research and government contexts [31,32]. More research at the policy level can inform and bridge the evidence-policy-practice gap [33] and can inform effective collaboration on the development of policy among academics, researchers and developers, health care planners and policy makers, practitioners, and consumer representatives [11,29,32].

Mental health policy and action documents have a critical role and responsibility for optimizing technology integration within the mental health care system. The impact of such a role is well illustrated by the policy example of Australia, a country that is at the forefront of integrating technology in the delivery of mental health services. In 2012, the Australian government’s Department of Health and Aging produced a policy document entitled, “E-Mental Health Strategy for Australia,” which provided a vision for e-mental health services and key areas for action. The strategy also provided guidance on investment and development to advance the field and was created through input from expert advisors representing top researchers within the field, consumer representatives, and executive leads of organizations delivering mental health services using technology [34].

The role of technology in mental health policy is even more critical considering the COVID-19 pandemic. Given this, it is important to understand the priority given to technology in government policy and to monitor the advancement of this priority at the policy level before, during, and after the pandemic. In 2017, a systematic review was published with the aim of comparing areas of focus on the use of technology in pediatric mental health care by examining research studies, and government and organizational documents. It included 2 Canadian-based policy documents, 1 at the national level, and 1 from Ontario [32]. However, to our knowledge, no systematic review has focused on the extent to which technology is discussed in mental health policies from all Canadian provinces and territories to date. Thus, the objective of this systematic policy review is to determine the extent to which technology is considered in Canadian-based mental health policy documents. Specifically, we aimed to synthesize the policy priorities addressed in government-based MHA documents and to better understand the extent to which technology is described as having a role in achieving these policy priorities.

## Methods

### Collection and Selection of MHA Policy Documents

**Figure 1:**
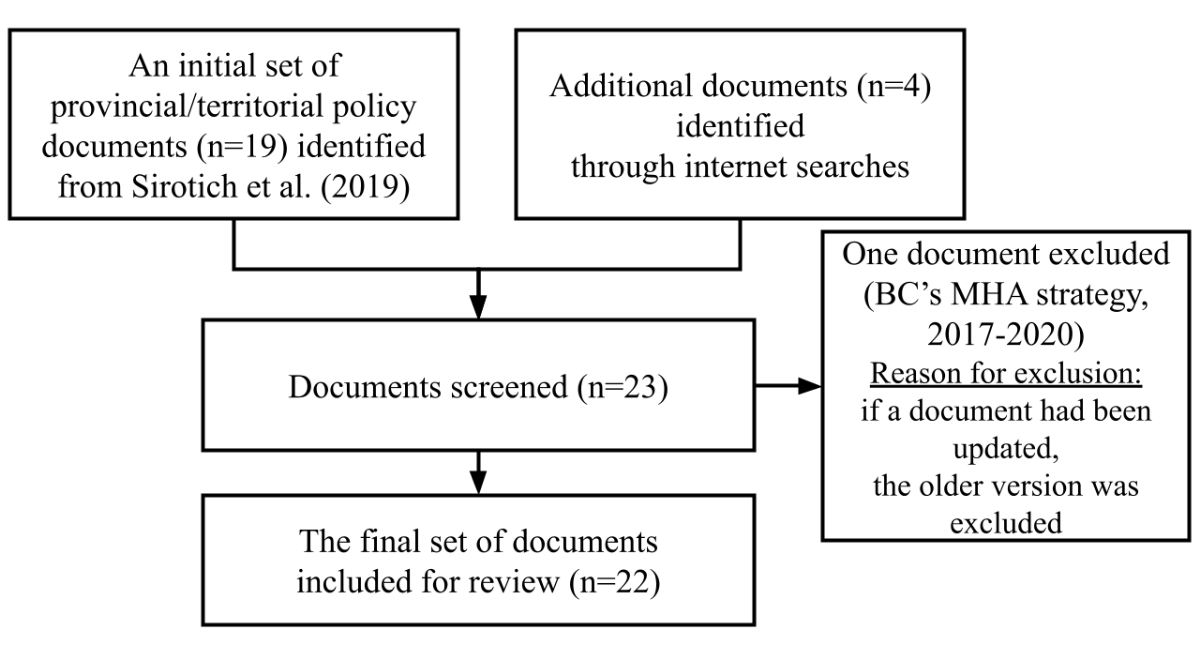
Identification and selection of MHA policy documents. MHA: mental health and addictions.

As illustrated in Figure 1, we started with an initial set of 19 provincial/territorial (P/T) policy documents published until June 2017 that were identified as part of a project led by CEA on the development of a pan-Canadian MHA performance measurement framework [2,35]. These initial documents were obtained from a systematic internet search of P/T government websites. They were then confirmed for completeness for each jurisdiction in consultation with researchers, data managers, and policy makers from the Mental Health Commission of Canada’s (MHCC) Provincial and Territorial Advisory Group and through consensus by the research team based on inclusion criteria pertaining to policy timeframes, focus on special populations, and versions of documents [2,35]. Two additional team members from this current review (SL and LS) considered each of these 19 documents to determine if they met our inclusion criterion for the current review and to assess the need for additional searches. The inclusion criterion was a document that describes a P/T vision or strategy for mental health service delivery; we also endeavored to ensure that each P/T was represented by a mental health policy document. In the exceptional case of Nunavut, which became the newest Canadian territory in 1999 at which time a vision or strategy document did not exist, the next closest type of document was included [36]. The exclusion criterion was any older document for which an updated version of the same type of document from that P/T was available. This was to provide the most updated perspective of mental health policy across the P/T at the time of conducting the review.

To identify updated versions of the policy documents, during the summer of 2019, we conducted internet searches (on the P/T websites and using the Google search engine). Based on this search, we identified 4 additional documents: (1) a recent MHA policy document from British Columbia (2019-2029) [37], (2) Nova Scotia’s progress update on the 5-year plan (2016) [38], (3) an action plan for suicide prevention in Nunavut (2016-2017) [39], and (4) the Final Report of Ontario’s MHA Leadership Advisory Council (2017) [40]. Thus, we had a total set of 23 documents, from which we excluded 1, BC’s MHA Strategy (2017-2020) [41] because a newer version was available; this resulted in a final set of 22 documents. Each of these documents is presented in Multimedia Appendix 1, including title, source in terms of P/T, URL links, and overview.

All documents described a vision or framework, strategy or action plan, or progress update related to MHA policy in that province/territory, 20 documents addressed MHA policies pertaining to the P/T’s population as a whole, while 2 documents were about MHA policy specific to a subpopulation in that P/T (ie, the 2013 BC First Nations and Aboriginal People’s MHA 10 Year Plan and the 2014 Yukon’s Child and Youth MHA Framework (2014) [42,43].

### Data Extraction and Analysis

We first reviewed the documents using an initial list of 9 technology words (*e-mental health*, *tech*, *tele*, *net*, *internet*, *web*, *virtual*, *app*, and *digital*) to gain a preliminary impression of how technology was considered in the government documents. These 9 words were based on SL and CEA’s previous review of the literature on technology in mental health care [12] and recent academic and gray literature (eg, e-mental health documentation from the MHCC [10]). We reviewed the documents systematically using the control-F function and recorded instances where the 9 words were mentioned in a Microsoft Word document table. We did not find many examples of policy documents mentioning the use of technology; however, we felt that perhaps this could have been due to the limited number of words we used to search the documents. The initial search process informed our understanding of the types of terms used in the policy documents and led to the development of a more detailed protocol to guide our review. Our protocol included data extraction tables for organizing the data (eg, name of policy document, year of publication, source, excerpts from the documents illustrating use of a technology term). As part of this protocol, we also created a process for validating the data extraction and analysis to enhance reliability of our results. Our final protocol included 39 technology-related keywords that would be used to examine the extent to which each policy document addressed the topic of technology in relation to the delivery of mental health services. This list was finalized through pilot testing in several of the documents before being implemented systematically across all the documents. We also translated this keywords list into French, first by a native French-speaking team member, with initial validation by a second native French-speaking team member (LS) and final validation by the project lead (SL) who is a native English-speaking team member. The keywords list in English and French is presented in Table 1.

Next, we reviewed each policy document to gain a better understanding of the type of document (ie, vision, strategy, and progress related to mental health policy). We determined the type of document mainly based on their titles, for example, documents that typically used the terms *vision*, *strategy*, or *update* in the titles. We also conducted a modified conventional content analysis [44] of the MHA policy priorities described in these documents to obtain a snapshot of the recurrent themes addressed across the set of 22 documents. This involved reviewing the content of each document with a focus on extracting the MHA priorities using a data extraction table in a Microsoft Excel file (LS). The priorities were then coded inductively (ie, staying close to the terms used) and then grouped to arrive at a list of 14 categories. These categories were then reviewed by 2 members of the team (SL and LS) to discuss overlaps, redundancies, and what each category represented in terms of content. This led to a final coding framework of 10 MHA priorities that were represented across the 22 documents. Finally, each of the documents was then re-examined by LS to tabulate which of the 10 MHA priorities was addressed by each of the P/Ts, and this tabulation was then validated by HL.

Next, using the control-F function, we searched each of the documents using the keyword terms listed in Table 1. All findings that represented the use of technology within the context of mental health care were charted in a Microsoft Excel file. The data in this Excel file were initially extracted by a member of our team (LS) and validated by another (HL).

After we completed the search for the keywords and all instances of the use of technology were extracted from the policy documents, we grouped the 39 keywords into 22 categories to facilitate the synthesis process. Specifically, we merged the synonyms and abbreviations of a keyword into the same category. For example, *electronic* and the abbreviation *EHR* (*electronic health record*) were grouped into the *electronic* category. We also removed keywords that had not been mentioned in any of the policy documents. For example, *EMR* (*electronic medical record*) was removed, since it was not mentioned in any document. This final set of 22 categories was arrived at through an iterative process involving discussion and consensus between SL and LS. We then organized our findings based on the number of policy documents (n) that mentioned each category out of the 22 policy documents (eg, the keyword category *Tele* was mentioned in 16 out of the 22 policy documents; n=16/22; Table 2); subsequently, we identified which P/Ts had documents mentioning each category (Table 2). We also counted the number of categories mentioned by each P/T out of the total 22 categories, as represented by n (eg, only 1 keyword category, *electronic*, was mentioned by the Northwest Territories; Multimedia Appendix 3). Note that we counted compound keywords twice to maintain a systematic approach to the keyword search of the policy documents. For example, *telephone* was counted once in *tele* and once in *phone*, and *text messages* was counted once in *text* and once in messag*.

**Table 1:** List of English and French keywords^a^ used to search the documents.

Keywords list (English)	Keywords list (French)
app (eg, application)	appli (eg, application)
artificial intelligence	intelligence artificielle
avatar	avatar
bots (eg, chatbots, chat bots, robots), chat (eg, live chat, live-chat)	bots (eg, robots), chat (eg, chatbots, chatter, chat en ligne, chat en direct), dialogueur, agent conversationnel
messag*, instant messaging	messagerie instantanée
cell, phone, line, link	cellulaire, téléphone
mobile (eg, mobile device, mobile health), mhealth	mobile (eg, appareil mobile, santé mobile) msanté (eg, m-Santé, m-santé)
text, SMS	message texte, texto, SMS (eg, message-texte)
smart (eg, smartphone, smart phone, smart watch, smartwatch)	intelligent (eg, téléphone intelligent, montre intelligente)
computer (eg, laptop computer, desktop computer, personal computer), laptop	ordinateur, portable (eg, ordinateur portable, ordinateur-portable)
cyber (eg, cyber-psychology, cyber psychology, cyberspace, cyber-space)	cyber (eg, cyberpsychologie, cyber-psychologie, cyber psychologie, cyber-espace, cyber espace)
device (eg, wearable device, wearable-device, mobile device, portable device)	appareil (eg, appareil portable)
digital	digital
e-mental health	santé mentale électronique, e-santé mentale, santé numérique, cybersanté mentale
email	courriel, courrier électronique
electronic, EMR^b^, EHR^c^, e-referral (eg, mail, electronic-mail, electronic case management, electronic-case-management, electronic medical record, electronic-medical-record, e-patient, e patient, electronic patient, electronic-patient, e-referral, e referral)	électronique, DME^d^ (eg, gestion de cas électronique, dossier médical électronique, patient électronique, référence électronique, référencement électronique)
net (eg, internet), web (eg, website)	net (eg, internet), toile, web (eg, site web)
online	en ligne
portals	portail
sensor	senseur
platform, (Headspace), (ACCESS^e^ Open Minds^f^)	plateforme, plate-forme
social media (eg, social-media)	médias sociaux, réseaux sociaux
tech (eg, technology, communication technology, health information and communication technology)	techno (eg, technologie, technologique, technologies de l’information et de la communication en santé)
tele (eg, telehealth, tele-health, tele-medicine, telemedicine, tele-mental-health, telementalhealth, tele-psychiatry, telepsychiatry, tele-psychology, telepsychology, tele-therapy, teletherapy)	télé (eg, télé-santé, télé santé, télé-médecine, télé médecine, télé santé mentale, télé-psychiatrie, télé psychiatrie, télé-psychologie, télé psychologie, télé-thérapie, télé thérapie)
video (eg, video-health, videohealth, video mental health, videomentalhealth, video-psychiatry, videopsychiatry, video-psychology, videopsychology, video-therapy, videotherapy)	vidéo (eg, vidéo-santé, vidéo santé, vidéo santé mentale, vidéo-psychiatrie, vidéo psychiatrie, vidéo-psychologie, vidéo psychologie, vidéo-thérapie, vidéo thérapie)
virtual (eg, virtual reality, virtual-reality), VR^g^	virtuel (eg, réalité virtuelle)

^a^The original list of 9 words included *e-mental health*, *tech*, *tele*, *net*, *internet*, *web*, *virtual*, *app*, and *digital*.

^b^EMR: electronic medical record.

^c^EHR: electronic health record.

^d^DME: dossier médical électronique.

^e^ACCESS: Adolescent/young adult, Connecting to, Community-driven, Early, Strengths-based and Stigma-free services.

^f^A national network connecting youth, families, caregivers, researchers, service providers, and policy makers to improve youth mental health care across Canada.

^g^VR: virtual reality.

**Table 2:** Frequency of technology-related categories mentioned in the provincial/territorial mental health and addictions policy documents.

Category	Examples of keywords^a^	Policy documents mentioning the category^b^, n	Province/territory mentioning the category (item #)^c^
Tele (*télé* in French)	telehealth, TeleHealth, tele-health, telephone, telepsychiatry, tele-psychiatry, telephone counselling, teleconferencing, telecounselling, telehealth network, telehealth consultation, video teleconferencing, tele-mental health, telemedicine, *consultation téléphonique psychosociale*	16	Alberta (2), British Columbia (3 and 4), Manitoba (5), Newfoundland and Labrador (7 and 8), Nova Scotia (9 and 10), Nunavut (13), Ontario (14 and 17), Prince Edward Island (18), Québec (19), Saskatchewan (20), Yukon (21 and 22)
Phone (*téléphone* in French)	phone, Kids Help Phone, telephone, telephone counselling, telephone screening, crisis phone lines, phone line, *téléphone*, phone-based coaching service	12	Alberta (1), Newfoundland and Labrador (7 and 8), Nova Scotia (9 and 10), Nunavut (13), Ontario (14), Prince Edward Island (18), Québec (19), Saskatchewan (20), British Columbia (4), Yukon (21)
tech/techno	technology-based services, technology-based solutions, technologies, information technology, communication technology, technology, e-health technology, technology-based interventions, communication technologies, technological solutions, distance technology, emerging technology, evolving technologies, *technologie*	11	Alberta (1 and 2), Manitoba (5), New Brunswick (6), Newfoundland and Labrador (7 and 8), Ontario (16 and 17), Québec (19), Saskatchewan (20), Yukon (22)
online	online counselling, online, online information, online cognitive behavioural therapy, online self-help supports, online training, online program, online options, online delivery, on-line resource, healthlineonline.ca, online gateways, provincial online hub, centralized online hub, online psychotherapy, ReachOut.com online platform, 211 online directory of community services (sk.211.ca), online and distance supports, online knowledge exchange (KE)^d^ space, online access point, online clinical treatments, online treatments, online resources, online directory, online tools, online modules, online innovation hub, online prevention, online clinical supports, online application, online chat capabilities, online experience, online government resources, online access, online peer support tools	10	Alberta (1), British Columbia (4), Newfoundland and Labrador (7 and 8), Nova Scotia (10), Nunavut (13), Ontario (14 and 15), Saskatchewan (20), Yukon (22)
line	distress lines, Mental Health Crisis Line, helpline, Provincial Warm Line, crisis line, Problem Gambling Helpline, Kamatsiaqtut Help Line, crisis phone lines, HealthLine (811), healthlineonline.ca, phone line, community-based crisis lines, Kids Help Line, crisis helpline, line, crisis lines network	10	Alberta (1), British Columbia (4), Newfoundland and Labrador (7 and 8), Nova Scotia (9 and 10), Nunavut (13), Saskatchewan (20), Yukon (21 and 22)
web	web-based, website, mental health website, youth mental health websites, web resource, web application	10	Alberta (1 and 2), British Columbia (4), Manitoba (5), New Brunswick (6), Newfoundland and Labrador (7 and 8), Ontario (14), Saskatchewan (20), Yukon (21)
virtual	virtual resources, virtual counselling, virtual solutions, virtually, virtual reality, virtual teams, virtual mental health counselling	6	Alberta (1 and 2), British Columbia (4), Newfoundland and Labrador (7 and 8), Yukon (22)
electronic	electronic health record, EHR^e^, electronically, electronic client record, electronic mental health information and support, electronic information and support, electronic links	6	Alberta (1), Newfoundland and Labrador (7), Northwest Territories (11), Ontario (14), Prince Edward Island (18), Yukon (21)
social media	social media, social media communications	5	British Columbia (3), Newfoundland and Labrador (7 and 8), Nunavut (13), Ontario (15)
video	video conference, videos, videoconferencing, video teleconferencing	4	Newfoundland and Labrador (7), Nova Scotia (9), Ontario (14), Saskatchewan (20)
app	mobile applications, app, application, online application, web application	4	Alberta (2), Nova Scotia (9), Yukon (21 and 22)
link	Health Link, links, electronic links	4	Alberta (1 and 2), Ontario (14), Yukon (21)
text	text, text messages	3	Newfoundland and Labrador (7 and 8), Nova Scotia (10)
e-mental health	e-mental health, e-mental health services, e-Mental Health and Addictions platform (eMHA^f^)	3	Newfoundland and Labrador (7 and 8), Saskatchewan (20)
platform	platform, MyHealth platform, mental health and addictions platform, e-Mental Health and Addictions platform (eMHA), central platform, integrated eMHA platform, online platform	3	Alberta (1 and 2), Saskatchewan (20)
net	internet, internet cognitive behavioural therapy, iCBT^g^	3	British Columbia (4), Newfoundland and Labrador (7), Saskatchewan (20)
chat	chat, online chat capabilities, web-based chat capabilities	3	British Columbia (4), Nova Scotia (10), Yukon (21)
digital	digital therapy, digital view	2	Alberta (1), Newfoundland and Labrador (7)
portal	portal, web-based portal	2	British Columbia (4), Ontario (14)
mobile	mobile applications	1	Alberta (2)
messag*	text messages	1	Nova Scotia (10)
e-referral	e-referral tool	1	Alberta (1)

^a^Examples of keywords for each category were systematically extracted from the policy documents reviewed.

^b^Compound keywords were counted twice to maintain a systematic approach to the keyword search of the policy documents. For example, *telephone* was counted once in *tele* and once in *phone*, and *text messages* was counted once in *text* and once in messag*.

^c^The item number in parentheses reflects the item number in Multimedia Appendix 1.

^d^KE: knowledge exchange.

^e^EHR: electronic health record.

^f^eMHA: e-Mental Health and Addictions platform.

^g^iCBT: internet cognitive behavioural therapy.

## Results

### Description of the MHA Policy Documents

Most Canadian P/T governments (including Manitoba, New Brunswick, Northwest Territories, Prince Edward Island, Québec, and Saskatchewan) had 1 MHA policy document [45-50]. Alberta [51,52], British Columbia [37,42], Newfoundland and Labrador [53,54], Nova Scotia [38,55], Ontario [40,56-58], and Yukon [43,59] had two or more MHA policy documents. Nunavut did not have any MHA policy documents; instead, a statement of overall government directions and an action plan for suicide prevention in Nunavut were used in the analysis [36,39]. The types of policy documents we reviewed were visions or frameworks, strategies or action plans, and progress updates. The majority (n=17/22) of the policy documents were focused on MHA policy strategies and action plans. The remaining 5 documents were about visions or frameworks for an MHA system (n=2/22), progress updates on the MHA strategies or action plans (n=1/22), or combined MHA strategies or action plans and progress updates (n=2/22). We extracted data on the overview of each MHA policy document, particularly focusing on P/T policy priorities addressed in the documents (see Multimedia Appendix 1).

Our synthesis of P/T MHA policy priorities identified 10 themes, with 7 themes being found in the policies of all 13 P/Ts: wellness/recovery/MHA promotion, prevention, and early intervention (eg, promoting good mental health, reducing stigma); service integration (eg, integration of service delivery to improve the currently fragmented system); collaboration (eg, promoting a multidisciplinary and collaborative team approach); children, youth, and families (eg, youth suicide prevention, strongest families program); improving access (eg, better access to services for remote, rural, and underserved communities; decreasing wait times and travel times); cultural safety and indigenous communities (eg, culturally relevant treatments and services, health equity for diverse populations); and use of technology (eg, sharing of evidence-based MHA information or services online)*.* The remaining 3 themes were support for seniors (n=12/13); innovation, improvement, and research (n= 2/13); and reducing bullying (n=6/13). As depicted in Multimedia Appendix 2, we organized the policy priorities as themes related to a population or a service; 3 of the priorities related to specific populations (children, youth, and seniors; and diversity of communities, such as indigenous communities, racialized groups, new arrivals, and persons with disability), and the remaining 7 were related to a service priority.

### Use of Technology in the MHA Policy Documents

Of the 39 keywords listed in Table 1, we identified 22 categories addressing the use of technology to deliver mental health services listed in Table 2. Table 2 also includes examples of keywords from each category that were used in the policy documents.

The 6 most common keyword categories were *tele* (n=16/22), *phone* (n=12/22), *tech* (n=11/22), *online* (n=10/22), *line* (n=10/22), and *web* (n=10/22). Among the 6 most common categories, 3 pertained to connecting people remotely in real-time: *tele* (eg, *telephone*, *telehealth*, *telepsychiatry*; n=16/22), *phone* (eg, *telephone counselling*, *crisis phone lines*, *phone-based coaching service;* n=12/22), and *line* (eg, *distress lines*, *mental health crisis line*, *helpline*; n=10/22). Policy documents also referred to *technology-based services* in general without detailing specific technologies. As such, *tech* (n=11/22) was the third most common category, followed by *online* (n=10/22), *line* (n=10/22), and *web* (n=10/22). Examples of words mentioned in this regard include *technology-based services*, *technology-based solutions*, and *technologies*; *online counselling*, *online information*, and *online cognitive behavioural therapy*; and *web-based*, *website*, and *mental health website*.

In this review, *high-tech* was used to describe technologies other than telephone-based services (eg, live chat on a website, the use of smartphone apps) and *low-tech* was used to describe telephone-based services (eg, crisis telephone help lines). High-tech categories were less common and included *virtual* (n=6/22), *electronic* (n=6/22), *social media* (n=5/22), *video* (n=4/22), *app* (n=4/22), and *link* (n=4/22). Examples of words mentioned in this regard include *virtual resources*, *virtual counselling*, and *virtual reality*; *electronic health record* and *electronically*; *social media* and *social media communications*; *video conference* and *videos*; *mobile applications*, *app*, and *online application*; and *Health Link*, *links*, and *electronic links*.

Other high-tech categories and broader approaches to the use of technology were mentioned at an even lower frequency: *text* (eg, *text*, *text messages*), *e-mental health* (eg, e*-mental health*, e-*mental health services*, e*-Mental Health and Addictions platform*), *platform* (eg, *platform*, *MyHealth platform*, *mental health and addictions platform*, *online platform*), *net* (eg, *internet*, *internet cognitive behavioural therapy*, [*iCBT*]), and *chat* (eg, *chat*, *online chat capabilities*, *web-based chat capabilities*) were each mentioned in 3 policy documents (n=3/22). *Digital* (eg, *digital therapy*, *digital view*) and *portal* (eg, *portal*, *web-based portal*) were each mentioned in 2 policy documents (n=2/22). *Mobile* (eg, *mobile applications*), messag* (eg, *text messages*), and *e-referral* (eg, *e-referral tool*) were each mentioned in 1 policy document (n=1/22). Overall, there was a diverse range of high-tech categories mentioned in the P/T policy documents but these were mentioned at a much lower frequency compared to the low-tech categories.

Additionally, as shown in Multimedia Appendix 3, there was a high level of variation in the extent to which the policy documents referred to the 22 categories across the 13 P/Ts of Canada: Alberta (n=14/22), Newfoundland and Labrador (n=14/22), Yukon (n=11/22), British Columbia (n=10/22), Ontario (n=10/22), Saskatchewan (n=10/22), Nova Scotia (n=9/22), Nunavut (n=5/22), Manitoba (n=3/22), Prince Edward Island (n=3/22), Québec (n=3/22), New Brunswick (n=2/22), and Northwest Territories (n=1/22).

As noted above, more than half of the technology categories were mentioned in the policy documents of Alberta and Newfoundland and Labrador. Alberta’s MHA action plans (2015, 2017-2020) emphasize developing and implementing a range of technology-based solutions (eg, websites, telehealth, mobile apps, virtual solutions) for MHA problems, and combining and enhancing resources that are already available (ie, telephone-based interventions such as Health Link and Kids Help Phone) [51,52]. For example, in its 2017-2020 MHA action plan, it states the following: “develop virtual, technology-based solutions to help people access tools, information and treatment to address addiction and mental health issues,” and “share information on websites, telehealth, mobile, mobile applications, and other technologies” [51]. Similarly, Newfoundland and Labrador’s vision for a renewed MHA system (2017) and MHA action plan (2017-2022) highlighted using a range of technologies (eg, including telehealth, videoconferencing, telephone crisis lines, online, text, virtual reality, social media, electronic health records, e-mental health) to achieve a full continuum of care and improve access to services [53,54].

Regarding the policy documents that mentioned only a few technology categories (eg, Québec, New Brunswick, and the Northwest Territories), Quebec’s action plan (2015-2020) mentioned developing new technologies to offer a network of youth-friendly MHA services, reiterated the availability of 24-7 psychosocial telephone consultations, and mentioned 2 institutions that aimed to promote mental health and new technologies: the Centre national d’excellence en santé mentale (CNESM) and the Service d’accueil, d’analyse, d’orientation et de référence des services sociaux généraux (AAOR) offering services over the phone [49]. In the New Brunswick Family Plan (2017), the only reference to technology was “information related to MHA treatment options are available to the public through various means, including web-based and print materials” [46]. The only mention of technology in the Northwest Territories strategic framework (2016-2021) pertained to electronic health records to promote the integration of service delivery [47]. For more details, refer to Table 2.

## Discussion

Our objective for this policy review was to determine the extent to which technology is prioritized in mental health and addictions (MHA) policy documentation. Given that policy is context-based, we focused on the Canadian context. The methods that we used in this review can help to inform future efforts to monitor the evolutionary role of technology in mental health policy in Canada and abroad. Our findings have implications for policy makers, health care leaders, and researchers working towards improving access and quality of mental health services.

### Principal Findings

Our key findings are as follows. First, although all the documents referred to the use of technology for MHA care, there was a high level of variation in the extent to which technology was addressed across the P/T documents and how it was addressed. For example, Newfoundland and Labrador’s MHA vision (2017) and action plan (2017-2022) described a variety of technology-based services (including telehealth, videoconferencing, telephone crisis lines, online, text, virtual reality, social media, electronic health records, e-mental health) to support access and continuum of care [53,54], whereas the strategic framework from the Northwest Territories (2016-2021) only mentioned electronic health records to promote the integration of service delivery [47], and Québec’s action plan (2015-2020*)* only mentioned using technologies to offer MHA services to youth and providing psychosocial telephone consultations to the general population [49]. Second, only a few of the documents referred to the use of “high-tech” solutions, such as virtual reality and apps, with more than half mainly describing “low-tech” solutions, such as telephone-based helplines and telephone consultations for families. Considering the limitations of telephone-based interventions compared to other formats, such as video (eg, the latter resulting in fewer treatment errors and greater diagnostic and decision-making accuracy) [60] and considering mainstream society’s increasing use of other formats of communication, such as texting, live chat, videoconferencing, and social media, addressing high-tech solutions in Canadian mental health policy is warranted. Third, there was inconsistency in the use of technology-related terminology across the documents, with the term *e-mental health* (the term used by the MHCC) [10,30] rarely mentioned. Fourth, there was limited strategic focus and guidance in the policy documents on how to move forward with the successful implementation of technology-enabled services within the public mental health care system.

It is important to note that the P/T policy documents reflect governmental priorities for MHA services and did not have the purpose of providing a complete perspective on the use of technology. Although it may not be the intention of the policy documents to provide an exhaustive list of initiatives, describing exemplary evidence-based initiatives (eg, onlinetherapyuser.ca) that have received government funding is warranted to provide guidance to local health authorities. Moreover, the variation that we identified across P/Ts on the degree of attention to technology in MHA care is noteworthy.

There are several implications of our findings. First, there is a need for strategic policy attention on the integration of technology in mental health; this requires addressing topics such as funding for implementation initiatives, infrastructure, regulation, capacity building, intersectoral collaboration, translation research, policy-research-practice partnerships, among others [11,12,29,32,33], as well as partnerships between researchers, practitioners, and policymakers. Advancement in these areas can be facilitated through interactions among policy makers and health services researchers specializing in the use of technology to deliver mental health care. Choi et al [61] provide several insights that can be helpful in this regard, including recommendations to incentivize collaboration among policy makers and scientists, use of knowledge brokers (to facilitate knowledge translation), and acknowledgment of the various issues affecting collaboration, including different languages and perspectives on evidence and different timeframes on achieving deliverables. Policy-making initiatives that facilitate the involvement of consumers, caregivers, and service is also important to consider.

Second, the inconsistency in the use of technology-related terms across the P/Ts is not surprising given the evolving nature of the field itself and inconsistencies in the research literature. Nonetheless, this poses challenges to conducting future policy reviews to monitor advancement in the field. To address this issue, a living document of terminology, that is updated yearly, could be made accessible to mental health policy makers to encourage a level of standardization in the language used to refer to the use of technology in mental health care.

Third, the inconsistency in the degree of attention on technology across the P/T documents may be a reflection that policy makers have limited knowledge on the evidence and potential of using technology in this field, highlighting the value for knowledge translation and collaborative initiatives among policy makers and researchers. It may also reflect the degree to which technology is prioritized at the front-line level of service delivery, raising the question of whether there is a lack of equity across the country in terms of the public’s access to a wide range of services and interventions delivered through technology. This lack of attention in policy can contribute to a fragmented and unregulated approach to using technology to deliver mental health services in the country. Translational research for policy development and implementation planning is needed to better understand viewpoints from users, developers, providers, and policy makers on the implementation of technology-supported innovations, and to monitor the impact of implementation in terms of cost-effectiveness, access, and engagement, and the integration of models such as stepped care and clinical staging [11,30].

Fourth, it will be important to monitor the extent to which rapid responses to delivering mental health care in the context of the COVID-19 pandemic to address the increased need for mental health support among the general population [62] and those with existing MHA conditions [63-66] are reflected in mental health policy. Several of the P/Ts have responded to COVID-19 by providing information to the public about COVID-19 and maintaining mental health via social media [67]; delivering cognitive behaviour therapy through text messaging with mental health professionals (eg, Alberta’s Text4Hope); expanding existing online mental health counseling programs (eg, British Columbia’s MindHealthBC); and expanding use of existing mental health services for children and youth, such as text, live chat, mobile apps, and phone-based services [68]. At the same time, it is unclear whether the rapidly deployed initiatives are sufficient for responding to the mental health impacts of COVID-19 and to what extent will these initiatives address the already existing gaps in the continuum of mental health care for those with chronic mental health conditions. Given this, there is a continued need for comprehensive policy and research to support a coordinated and evidence-based integration of technology in the Canadian mental health care system, during and after the COVID-19 pandemic.

Fifth, there is a further need for P/Ts and national collaboration to develop reliable and comprehensive environmental scans of the implementation of technology in Canadian mental health services, including analysis of failed attempts. Sixth, the development of a pan-Canadian framework for action that addresses the integration and coordination of technology in mental health service delivery can also help to guide P/T initiatives in this field.

Sixth, our findings provide a prepandemic baseline and replicable methods to inform a subsequent study of how the use of technology-supported services and innovations emerge as an important priority in mental health policy and practice, during and after the COVID-19 pandemic. Future research can also focus on examining how technology is considered in relation to the various mental health priorities addressed by policy documents (eg, use of technology in promoting wellness/recovery/MHA promotion, prevention, and early intervention; use of technology in promoting collaborative care; and use of technology in senior’s mental health).

### Limitations

We used an existing database (ie, the 19 P/T policy documents included in Sirotich et al [2]) as the main source of policy documents for our review. Therefore, it is possible that we might have missed some relevant documents. It is also acknowledged that there may be other documents potentially relevant to this review in these jurisdictions, but the documents we have identified outline government priorities for MHA care at the highest level and therefore are of primary importance for communicating a vision and strategy for mental health care services within a government’s jurisdiction. It is also acknowledged that policies are frequently replaced; this review provides a report of relevant content available as of June 2019. In addition, we did not read through each page of each of the 22 documents to identify descriptions on the use of technology; although it was considered, it was deemed unfeasible by our team and also a potentially less reliable method of validation. We also believe that the final list of words we developed is quite comprehensive in its ability to capture the use of technology in mental health service delivery in these policy documents and that our methodology is both feasible and can be reliably replicated in future updated reviews.

### Conclusions

This review on Canadian P/T mental health policy documents suggests that despite the potential for technology to improve access to and quality of MHA services, its implementation has been fragmented to date. Specifically, we identified large variations across Canadian P/T policy documents in terms of the types of technologies mentioned. Furthermore, many of the P/T policy documents mentioned only low-tech solutions, such as telephone-based interventions. The review also shows the limited attention given in the mental health policy documents to the evidence-based literature on the use of technology to deliver mental health services, suggesting a potential gap in knowledge on the part of policy makers regarding the evidence in this field. This gap can be reduced through collaborative interactions among academics, policy makers, and practitioners facilitated through sectors of P/T governments (eg, research and innovation, health services). It would be worth considering repeating this review at regular intervals, which would document the advancement of technology in care delivery, including related responses to the COVID-19 pandemic.

## Appendix

Multimedia Appendix 1 Overview of mental health policy documents reviewed.

Multimedia Appendix 2 Recurrent themes identified in mental health and addictions (MHA) policy priorities across the 13 Canadian provinces and territories.

Multimedia Appendix 3 Frequency of technology-related categories mentioned by each province or territory.

## Abbreviations

AAOR: Service d’accueil, d’analyse, d’orientation et de référence des services sociaux généraux
CNESM: Centre national d’excellence en santé mentale
EHR: electronic health record
EMR: electronic medical record
ICT: information and communication technology
iCBT: internet cognitive behavioural therapy
MHA: mental health and addictions
MHCC: Mental Health Commission of Canada’s
P/T: provincial/territorial
