# Supplementary material for: Priority Given to Technology in Government-Based Mental Health and Addictions Vision and Strategy Documents: Systematic Policy Review
Source: J Med Internet Res. 2021 May 5;23(5):e25547. doi: 10.2196/25547 (PMC8135019; doi:10.2196/25547)
Supplement: Multimedia Appendix 1 [file jmir_v23i5e25547_app1.docx]

**Multimedia Appendix 1.** Overview of mental health policy documents reviewed.^a^

| Item # | Province /  Territory | Title of Policy Document and URL Link^b^ | Policy Document Overview |
| --- | --- | --- | --- |
| **1** | AB | Valuing Mental Health: Report of the Addictions and Mental Health Review Committee. 2015.  [**https://open.alberta.ca/dataset/d8413604-15d1-4f15-a979-54a97db754d4/resource/1a5e7a16-3437-428e-b51f-4ba9201767a4/download/alberta-mental-health-review-2015.pdf**](https://open.alberta.ca/dataset/d8413604-15d1-4f15-a979-54a97db754d4/resource/1a5e7a16-3437-428e-b51f-4ba9201767a4/download/alberta-mental-health-review-2015.pdf) | [Extracted from p.2]  A whole-of-government approach aimed at making services more person-centered, with more emphasis on promotion of good mental health, early intervention, treatment of mental illness as a chronic disease, using multidisciplinary teams and better coordination, flexibility in access, information sharing and use of evidence.  This document highlights 4 priorities to address:  1. Develop an integrated service delivery system for addiction and mental health by harmonizing the roles and goals of Alberta Health and Alberta Health Services;  2. Progress towards achieving a person-centered system for addiction and mental health;  3. Reduce distress, suffering, and health care costs by allowing primary health care providers to play a stronger role in the screening, prevention, early intervention, and continuity of care of addiction and mental health;  4. Achieve better access, quality, and efficiency in addiction and mental health services by establishing a leadership team to implement this report.  **Timeline: Not mentioned** |
| **2** | AB | Valuing Mental Health: Next Steps. 2017.  [**https://open.alberta.ca/dataset/25812976-049c-43c9-9494-77526c6f6ddd/resource/684600a3-a0ea-440c-a053-38a4cef83de9/download/alberta-mental-health-review-next-steps-2017.pdf**](https://open.alberta.ca/dataset/25812976-049c-43c9-9494-77526c6f6ddd/resource/684600a3-a0ea-440c-a053-38a4cef83de9/download/alberta-mental-health-review-next-steps-2017.pdf) | [Extracted from pp.4-6 & p.20]  A whole-of-government approach aimed at making services more person-centered, with more emphasis on promotion of good mental health, early intervention, treatment of mental illness as a chronic disease, using multidisciplinary teams and better coordination, flexibility in access, information sharing and use of evidence  "Initial efforts will target four populations requiring immediate attention:  • Children, youth and families;  • People with multiple and complex needs;  • Individuals requiring addiction services; and  • Indigenous people and communities" (p.6)  **Timeline: 2017 –** **2020** for the 18 actions building on activities underway and the 10 remaining recommendations adopted in 2015 |
| **3** | BC | A Path Forward: BC First Nations and Aboriginal People’s Mental Wellness and Substance Use—10 Year Plan. 2013.  [**https://www.fnha.ca/Documents/FNHA_MWSU.pdf**](https://www.fnha.ca/Documents/FNHA_MWSU.pdf) | [Extracted from p.14. & pp.25-32]  "MENTAL WELLNESS AND SUBSTANCE USE STRATEGIC DIRECTIONS AND ACTIONS (4 Action Plans)  1. Holistic Wellness: All First Nations and Aboriginal [Indigenous] people in BC  2. Community Care: First Nations and Aboriginal [Indigenous] people with vulnerabilities that compromise wellness  3. Integrated Care: First Nations and Aboriginal [Indigenous] people with moderate needs that compromise wellness  4. Specialized Care: First Nations and Aboriginal [Indigenous] People with Complex Needs that compromise wellness" (pp.25-32)  “Goals:  1. To improve services, supports, and health outcomes for all First Nations and Aboriginal [Indigenous] people in BC.  2. To keep First Nations and Aboriginal [Indigenous] people’s well-being at the center of our initiatives, while maintaining a high operational standard, and cross-sectoral integration.  3. To ensure that mental wellness and substance use strategies and actions for First Nations and Aboriginal [Indigenous] people reflect individual and family needs and are Community-driven and Nation-based.  4. To engage First Nations and Aboriginal [Indigenous] people in the journey towards improving health outcomes." (p.14)  **Timeline: 2013 –** **2023 (10-year plan)** |
| **4** | BC | A Pathway to Hope: A roadmap for making mental health and addictions care better for people in British Columbia. Strategy. 2019 – 2029.  [**https://www2.gov.bc.ca/assets/gov/british-columbians-our-governments/initiatives-plans-strategies/mental-health-and-addictions-strategy/bcmentalhealthroadmap_2019web-5.pdf**](https://www2.gov.bc.ca/assets/gov/british-columbians-our-governments/initiatives-plans-strategies/mental-health-and-addictions-strategy/bcmentalhealthroadmap_2019web-5.pdf) | [Extracted from p.3 & pp.12-15]  This document describes the government's 10-year vision for mental health and addictions in BC as well as short-term actions that will be taking place in the next three years to address current MHA issues.  “4 pillars:  1. Wellness promotion and prevention  2. Seamless and integrated care  3. An equitable access to culturally-safe and effective care  4. Indigenous health & wellness” (pp.12-15)  **Timeline: 2019** – **2029** (10-year vision) |
| **5** | MB | Rising to the Challenge: A strategic plan for the mental health and well-being of Manitobans. 2011.  [**https://www.gov.mb.ca/health/mh/docs/challenge.pdf**](https://www.gov.mb.ca/health/mh/docs/challenge.pdf) | [Extracted from pp.2-3]  Vision, Mission and Key Pillars. Uses Keyes (2005) dual continua model. MH Promotion; Recovery, Inclusion, Shared Responsibility, Leading and Promising Practices, Cultural Safety. 6-point strategic plan, each has Goals with multiple Objectives and Strategic Actions. More recently a comprehensive mental health and addictions strategy was recommended after a consultant study, which may lead to a new Policy.  "This 6-point strategic plan is aimed at improving the mental health of Manitobans by addressing:   - Mental health and well-being; - Access to services; - Innovation and research; - Social inclusion; - Family participation; and - Workforce development" (p.3)   **Timeline: 2011** – **2016** (5 years) |
| **6** | NB | New Brunswick Family Plan: Supporting Those with Addictions and Mental Health Challenges. 2017.  [**https://www2.gnb.ca/content/dam/gnb/Departments/eco-bce/Promo/family_plan/mentalhealth.pdf**](https://www2.gnb.ca/content/dam/gnb/Departments/eco-bce/Promo/family_plan/mentalhealth.pdf) | [Extracted from pp.3-5]  NB has a Family Plan which is related to an Economic Growth Plan and an Education Plan. This document is a subplan of the Family Plan. The global goal of this plan is to improve the social determinants of health. Expanding successful addiction and mental health promotion and prevention initiatives throughout the province. Improving community-based treatment options for those suffering from addictions and serious mental illness, such as implementing Supervised Community Care across the province (with an additional set of priorities/actions).  "The seven pillars of the New Brunswick Family Plan are:  1. Improving access to primary and acute care  2. Promoting wellness  3. Supporting those with addictions and mental health challenges  4. Fostering healthy aging and support for seniors  5. Advancing women’s equality  6. Reducing poverty  7. Providing support for persons living with a disability" (p.3)  **Timeline: Not mentioned** |
| **7** | NL | Towards Recovery: A Vision for a Renewed Mental Health and Addictions System for Newfoundland & Labrador. 2017.  [**https://www.health.gov.nl.ca/health/all_party_committe_report.pdf**](https://www.health.gov.nl.ca/health/all_party_committe_report.pdf) | [Extracted from pp.1-2 & p.15]  Report of an all-party Committee.  5 themes:  1. Improved mental health and addictions (MHA) promotion/prevention  2. Better access to more services  3. Better quality of care  4. Improved policy and programming  5. Strengthened community supports  The Action Plan includes 54 recommendations for a structure and process to implement the above, "i.e. the recommendations in this report support the implementation of an integrated, person-centered and recovery-focused system that provides the right care, at the right time and in the right place." (p.15)  **Timeline: Not mentioned** |
| **8** | NL | The Way Forward: Towards Recovery: The Mental Health and Addictions Action Plan for Newfoundland & Labrador. 2017.  [**https://www.gov.nl.ca/hcs/files/mentalhealth-committee-mentalhealth-pdf-mentalhealth-addictions-plan.pdf**](https://www.gov.nl.ca/hcs/files/mentalhealth-committee-mentalhealth-pdf-mentalhealth-addictions-plan.pdf) | [Extracted from p.2 & p.11]  The goal of this plan is to guide the implementation of the recommendations outlined in the previous doc - Towards Recovery and provide direction for mental health and addictions policy and programs from 2017 to 2022.  Four pillars set the policy direction for the mental health and addictions system:  1. Promotion, prevention and early intervention  2. Focusing on the person  3. Improving service access, collaboration and continuity of care  4. Including all people everywhere  **Timeline: 2017** – **2022** (5 years) |
| **9** | NS | Together We Can: The Plan to Improve Mental Health and Addictions Care for Nova Scotians. 2012.  [**https://novascotia.ca/dhw/mental-health/reports/Mental-Health-and-Addictions-Strategy-Together-We-Can.pdf**](https://novascotia.ca/dhw/mental-health/reports/Mental-Health-and-Addictions-Strategy-Together-We-Can.pdf) | [Extracted from p.5 & pp.11-30]  Nova Scotia’s Plan focuses on screening, early intervention (especially for children and youth), school-based supports (Schools Plus), shorter wait times, reducing stigma, cultural safety, community supports, services to meet the needs of Indigenous and diverse communities, and closing the gaps to ease transition between programs and services. It also emphasizes the need for supportive housing.  Vision: The outcome that is expected from this plan is better care sooner for Nova Scotians. Children and youth are a priority.  **Timeline: 2012** – **2017** (5 years) |
| **10** | NS | Together We Can: Progress Update January. 2016.  [**https://novascotia.ca/dhw/mental-health/reports/ProgressReport-Jan2016.pdf**](https://novascotia.ca/dhw/mental-health/reports/ProgressReport-Jan2016.pdf) | [Extracted from pp.6-7]  Nova Scotia’s Plan focuses on screening, early intervention (especially for children), school-based supports, shorter wait times, reducing stigma, cultural safety, community supports, and services to meet the needs of Indigenous and diverse communities. It also emphasizes the need for supportive housing.  **Timeline: progress update on the 5-year plan** |
| **11** | NT | Mind and Spirit: Promoting Mental Health and Addictions Recovery in the NWT: Strategic Framework.  2016 – 2021  [**https://www.hss.gov.nt.ca/sites/hss/files/resources/mind-spirit-promoting-mental-health-addictions-recovery-nwt-strategic-framework-web.pdf**](https://www.hss.gov.nt.ca/sites/hss/files/resources/mind-spirit-promoting-mental-health-addictions-recovery-nwt-strategic-framework-web.pdf) | [Extracted from pp.2-3 & p.19]  Four Key Directions:  1. Prevention and Early Intervention: “priority groups for prevention and early intervention will be children, youth, families, and elders” (p.2)  2. Recovery-Oriented System: "recovery is about being able to live a meaningful and satisfying life, whether or not there will continue to be ongoing symptoms or limitations caused by mental health problems, illnesses, or addictions" (p.2)  3. Personal Experience and Outcomes: "learn from individual experiences through ongoing research, monitoring and evaluation work, and improving the services we provide based on these lessons" (p.3)  4. Whole of Government Approach: "better integration of services between various levels of government and across departments" (p.3). For example, this approach can be achieved through electronic health records.  As of mid-2017 there was also a DRAFT Action Plan for Children and Youth with the same four key directions.  **Timeline: 2016** – **2021** (5 years) |
| **12** | NU | Sivumut Abliuqta Stepping Forward Together. 2014 – 2018.  [**https://www.gov.nu.ca/sites/default/files/sivumut_abluqta_-_eng.pdf**](https://www.gov.nu.ca/sites/default/files/sivumut_abluqta_-_eng.pdf) | [Extracted from pp.3-7]  Nunavut does not currently have a mental health plan or strategy, but they have created the Nunavut Suicide Prevention Strategy Action Plan and they have a statement of overall Government directions: Sivumut Abliuqta Stepping Forward Together 2014-2018 that has a strong emphasis on physical and mental health and reducing addictions and suicide.  This document refers to Nunavut's vision, people, land, economy, territorial government, etc.  **Timeline: 2014** – **2018** |
| **13** | NU | RESILIENCY WITHIN An action plan for suicide prevention in Nunavut. 2016 – 2017.  [**https://www.gov.nu.ca/sites/default/files/resiliency_within_eng.pdf**](https://www.gov.nu.ca/sites/default/files/resiliency_within_eng.pdf) | [Extracted from pp.4-16]  After an inquest into suicide was called in Nunavut January 2014, the jury produced 89 recommendations in their verdict.  This document, consistent with jury's verdict, is organized around 8 commitments to prevent suicide:  1. Focused and active approach  2. Continuum of mental health services  3. Equip youth to cope with adversity  4. Training  5. Research for understanding  6. Communicate with Nunavummiut  7. Early childhood development  8. Support community development  **Timeline: 2016** – **2017** (1 year) |
| **14** | ON | Open Minds, Healthy Minds: Ontario’s Comprehensive Mental Health and Addictions Strategy. 2011.  [**http://www.health.gov.on.ca/en/common/ministry/publications/reports/mental_health2011/mentalhealth_rep2011.pdf**](http://www.health.gov.on.ca/en/common/ministry/publications/reports/mental_health2011/mentalhealth_rep2011.pdf) | [Extracted from p.4 & pp.10-24]  Ontario’s Strategy focuses on mental health promotion (especially in schools and the workplace), early identification and intervention, and expanding mental health services, especially for children, youth, and youth transitioning to adult services. As of mid-2017, OMHM was still in effect, and annual and special reports of Ontario’s Mental Health and Addictions Leadership Advisory Council under the title ‘Better Mental Health Means Better Health Moving Forward’ have also been tabled.  4 goals:  "1. Improve mental health and well-being  2. Create healthy, resilient, inclusive communities  3. Early identification and intervention  4. Provide timely, high quality, integrated, person-centered health and other human services" (p.4)  Emphasis is placed on children and youth.  **Timeline: Not mentioned** |
| **15** | ON | Better Mental Health Means Better Health: 2015. Annual Report of Ontario’s Mental Health & Addictions Leadership Advisory Council.  [**http://www.health.gov.on.ca/en/common/ministry/publications/reports/bmhmbh/mental_health_adv_council.pdf**](http://www.health.gov.on.ca/en/common/ministry/publications/reports/bmhmbh/mental_health_adv_council.pdf) | [Extracted from p.2]  According to the document, council priorities (action plans): "(1) prevention, promotion and early intervention; (2) youth addictions; (3) supportive housing (4) system alignment and capacity; and (5) community mental health and addictions funding reform" (p.2)  5 initial recommendations:  "1) Implement the necessary policy and funding changes to make it easier for young people to transition from mental health and addictions services for youth to services intended for young adults  2) Implement a rigorous quality improvement strategy for both the community and hospital mental health and addictions sectors  3) Respond to the mental health impacts of inter-generational trauma in First Nation, Métis, Inuit, and urban Aboriginal [Indigenous] communities  4) Make it a priority to invest in supportive housing for people with mental illness and addictions  5) Assign responsibility for youth addictions to a single provincial ministry to facilitate age-appropriate, integrated programming" (p.2)  **Timeline: 2015 (1 year)** |
| **16** | ON | Moving Forward: Better Mental Health Means Better Health: 2016. Annual Report of Ontario’s Mental Health & Addictions Leadership Advisory Council.  [**http://www.health.gov.on.ca/en/common/ministry/publications/reports/bmhmbh_2016/moving_forward_2016.pdf**](http://www.health.gov.on.ca/en/common/ministry/publications/reports/bmhmbh_2016/moving_forward_2016.pdf) | [Extracted from pp.2-5]  4 guiding principles essential to a person-centered system: "a system that is equitable, accessible, high-performing and recovery-oriented" (p.2)  Since 2015, progress is being made in the following fields: youth, supportive housing, quality improvement, Indigenous partners  Importance of equity - want to achieve excellent MHA services and supports for all - especially racialized groups, people with disabilities, immigrants, refugees, homeless and LGBTQ people, French-speaking communities, Indigenous peoples  “Our recommendations for 2016, summarized below, have been carefully measured against three strategic considerations. 1. Promote, prevent, and intervene early 2. Close critical service gaps 3. Build foundations for system transformation” (p.5)  **Timeline: 2016 (1 year)** |
| **17** | ON | Mental Health and Addictions Realizing the Vision Better Mental Health Means Better Health: 2017. Final Report of Ontario’s Mental Health & Addictions Leadership Advisory Council.  [**http://www.health.gov.on.ca/en/common/ministry/publications/reports/bmhmbh_2017/vision_2017.pdf**](http://www.health.gov.on.ca/en/common/ministry/publications/reports/bmhmbh_2017/vision_2017.pdf) | [Extracted from pp.3-15]  In this final report, “Over the course of its three-year mandate, the Mental Health and Addictions Leadership Advisory Council has focused its efforts on fulfilling the four overarching objectives of Ontario’s Comprehensive Mental Health and Addictions Strategy, Open Minds Healthy Minds:  1. Improve mental health and well-being for all Ontarians;  2. Create healthy, resilient, inclusive communities;  3. Identify mental health and addictions problems early and intervene;  4. Provide timely, high quality, integrated, person-directed health and other human services.” (p.3)  2017 recommendations  1.THE SYSTEM: Meeting the Needs of Ontarians  - Promote, prevent and intervene early; adopt 5in5 THRIVE; the mental health promotion, prevention, early intervention action framework  - Close critical service gaps; continue to address chronic gaps in youth addictions, psychotherapy and supportive housing  - Build foundations for system transforamtion; take action toward enhanced governance, equitable access to services, and youth policy reform  2.THE APPROACH: Critical Conditions for Success  - Health equity for diverse populations (Indigenous, racialized, immigrant, refugee, LGBTQ, low-income, Francophone, etc.)  - Wellness and recovery  - Addressing the needs of Indigenous communities and populations  - French language services  - Funding  3.THE PATH FORWARD: Future Considerations  - Pursue further study of, and address, additional areas of concern (including considering the needs of older adults/seniors, individuals with a developmental disability, Indigenous populations, and establishing greater MHA literacy within and between the primary care system and the MHA system)  **Timeline: 2017 (1 year)** |
| **18** | PE | Moving Forward Together: Mental Health and Addiction Strategy.  2016 – 2026.  [**https://www.princeedwardisland.ca/sites/default/files/publications/peimentalhealthaddictionsstrategy_moving_forward.pdf**](https://www.princeedwardisland.ca/sites/default/files/publications/peimentalhealthaddictionsstrategy_moving_forward.pdf) | [Extracted from pp.4-5]  This document sets out general priorities and notes that an Action plan for the first two years (2016-2018) is in the works. The approach is noted to be cross-Ministry/whole of Government and full continuum from promotion through recovery.  "5 interconnected strategic priorities:  1. Mental health promotion for people of all ages  2. Access to the right service, treatment, and support  3. An innovative and collaborative workforce  4. Invest early - focus on children, young people and families  5. Foster recovery and well-being for people of all ages" (p.5)  Emphasis on eliminating stigma and discrimination, improving the mental health of specific diverse populations, and increasing collaboration within government and between government and community.  **Timeline: 2016** – **2026** |
| **19** | QC | Faire Ensemble et Autrement Plan D’Action en Sante Mentale.  2015 – 2020.  [**http://publications.msss.gouv.qc.ca/msss/fichiers/2017/17-914-17W.pdf**](http://publications.msss.gouv.qc.ca/msss/fichiers/2017/17-914-17W.pdf) | [Extracted from the Table of Contents]  Full scope plan includes addictions and social determinants of health. A strong ‘rights’ approach.  Four areas of action:  1. Promote the primacy of the individual and the full exercise of citizenship;  2. Provide youth-friendly care and services from birth to adulthood;  3. Promote clinical and management practices that improve the care experience;  4. Ensure the performance and continuous improvement of mental health care and services.  **Timeline: 2015** – **2020** |
| **20** | SK | Working Together for Change: A 10-Year Mental Health and Addictions Action Plan for Saskatchewan. 2014.  [**https://www.saskatchewan.ca/government/health-care-administration-and-provider-resources/saskatchewan-health-initiatives/mental-health-and-addictions-action-plan**](https://www.saskatchewan.ca/government/health-care-administration-and-provider-resources/saskatchewan-health-initiatives/mental-health-and-addictions-action-plan) | [Extracted from pp.10-16]  Saskatchewan’s plan emphasizes an increased access to care, recovery-orientated care, prevention and early intervention, person and family-centered services, integrated and coordinated services, reducing stigma, providing services for diverse needs, and partnering with Indigenous, First Nations, Inuit and Métis (FNIM) to provide services.  Specific system goals:  "1. Enhance access and capacity and support recovery in the community  2. Prevention and early intervention  3. Create person and family-centered and coordinated services  4. Respond to diversities  5. Partner with First Nations and Métis peoples  6. Reduce stigma and increase awareness  7. Transform the system and sustain change" (see Table of Contents)  **Timeline: 2014** – **2024** (10-year plan) |
| **21** | **YT** | A Child and Youth Mental Health and Addictions Framework for the Yukon. 2014.  [**https://yawc.ca/files/a-child-youth-mental-health-addictions-framework-for-yukon-2014.pdf**](https://yawc.ca/files/a-child-youth-mental-health-addictions-framework-for-yukon-2014.pdf) | [Extracted from p.v & p.4]  The framework:  - Deals with meeting the mental health and addictions needs of children and youth  - Lays out a vision for a comprehensive continuum of mental health and addictions care programming across the domains of promotion, prevention, treatment & ongoing care, and research & evaluation tailored to the Yukon context  - Recognizes the importance of promotion, prevention and evidence-based care provision  - Adopts an integrated approach to mental health and substance use disorders  - Has several components: core values (child, youth and family-centered; integrated/coordinated; aligned and accessible; builds capacity; evidence-based and accountable), common language, mental health needs, comprehensive programming, service delivery model  - Emphasizes the need for collaboration across government departments, First Nations communities, agencies, and the private sector  - Allows children, youth and their families to receive care in their home communities to the greatest extent possible; emphasizes access and reach of specialized services for children and youth with more complex needs  **Timeline: Not mentioned** |
| **22** | **YT** | Forward Together: Yukon Mental Wellness Strategy. 2016 – 2026.  [**https://yawc.ca/files/forward-together-yukon-mental-wellness-strategy-2016-26.pdf**](https://yawc.ca/files/forward-together-yukon-mental-wellness-strategy-2016-26.pdf) | [Extracted from p.iv &1]  Uses the WHO definition of mental health and includes substance use/misuse/abuse in the plan. Whole of Government approach and broad stakeholder responsibility.  "The primary objective of the Mental Wellness Strategy will be to increase seamless mental health, trauma and substance use services with equitable access; a full continuum approach that spans the lifetime; cascading and need-appropriate service delivery and service matching; and to be accessible through any entry point or provider in the Yukon system in a culturally competent manner." (p.1)  4 strategic priorities:  "1. Promotion and prevention  2. Service delivery  3. System performance and access  4. Innovation and research" (p.1)  **Timeline: 2016** – **2026** |

^a^There was 1 policy document reviewed by Sirotich et al. (2019) that we did not include in this policy review: Government of British Columbia. *B.C.’s Mental Health and Substance Use Strategy: 2017-2020.* Government of British Columbia; 2017. Available at: https://www.health.gov.bc.ca/ library/publications/year/2017/mental-health-substance-use-strategy.pdf. Accessed August 15, 2018., as it met our exclusion criteria [ie, if the document was updated, we only included the updated version (see Item 4)].

^b^The links to these documents were checked and updated on February 25, 2021.
