# Supplementary material for: Priority Given to Technology in Government-Based Mental Health and Addictions Vision and Strategy Documents: Systematic Policy Review
Source: J Med Internet Res. 2021 May 5;23(5):e25547. doi: 10.2196/25547 (PMC8135019; doi:10.2196/25547)
Supplement: Multimedia Appendix 2 [file jmir_v23i5e25547_app2.docx]

**Multimedia Appendix 2.** Recurrent themes identified in mental health and addictions (MHA) policy priorities across the 13 Canadian provinces/territories.

| Theme | Province/territory | | | | | | | | | | | | |
| --- | --- | --- | --- | --- | --- | --- | --- | --- | --- | --- | --- | --- | --- |
|  | Alberta | British Columbia | Manitoba | New Brunswick | Newfoundland and Labrador | Nova Scotia | Northwest Territories | Nunavut | Ontario | Prince Edward Island | Québec | Saskatchewan | Yukon |
| **Wellness/Recovery/Mental health and addictions (MHA**^a^**) promotion, prevention, and early intervention**  eg, promoting good mental health, promoting well being, reducing distress and suffering, reducing stigma/raising awareness | ✔ | ✔ | ✔ | ✔ | ✔ | ✔ | ✔ | ✔ | ✔ | ✔ | ✔ | ✔ | ✔ |
| **Service integration**  eg, developing an integrated service delivery system for addiction and mental health/filling in current gaps of fragmented system/continuum of services, enhancing integrated healthcare, integration of MHA^a^ services | ✔ | ✔ | ✔ | ✔ | ✔ | ✔ | ✔ | ✔ | ✔ | ✔ | ✔ | ✔ | ✔ |
| **Collaboration**  eg, multidisciplinary team approach, reducing duplication (eg, electronic health records), improving efficiency of MHA^a^ services | ✔ | ✔ | ✔ | ✔ | ✔ | ✔ | ✔ | ✔ | ✔ | ✔ | ✔ | ✔ | ✔ |
| **Children, youth, and families**  eg, youth mental health, strongest families program, youth suicide prevention, LGBTQ2S+^b^ youth, behavioral problems, depression and anxiety, early identification and prevention, schools, young parents, underserved youth, psychosis | ✔ | ✔ | ✔ | ✔ | ✔ | ✔ | ✔ | ✔ | ✔ | ✔ | ✔ | ✔ | ✔ |
| **Improve access**  eg, better access to services for remote, rural, and underserved communities, provision of health services in prisons (#7)^c^, enhancing after-hour supports (#20, #22)^c^, decreasing wait times/travel times | ✔ | ✔ | ✔ | ✔ | ✔ | ✔ | ✔ | ✔ | ✔ | ✔ | ✔ | ✔ | ✔ |
| **Cultural safety/Indigenous communities**  eg, cultural and traditional knowledge, culturally relevant treatments and services, discrimination, social inclusion/community-based services, health equity for diverse populations (eg, Indigenous communities, racialized groups, new arrivals, persons with disabilities) | ✔ | ✔ | ✔ | ✔ | ✔ | ✔ | ✔ | ✔ | ✔ | ✔ | ✔ | ✔ | ✔ |
| **Use of technology**  eg, sharing of evidence-based MHA^a^ information/services online (#6)^c^ | ✔ | ✔ | ✔ | ✔ | ✔ | ✔ | ✔ | ✔ | ✔ | ✔ | ✔ | ✔ | ✔ |
| **Support for seniors**  eg, senior’s mental health, housing for seniors (#1)^c^, transitioning to seniors (#1)^c^, better meeting the needs of elders (#3)^c^ | ✔ | ✔ | ✔ | ✔ | ✔ | ✔ | ✔ |  | ✔ | ✔ | ✔ | ✔ | ✔ |
| **Innovation, improvement & research**  eg, encouraging research and innovation, research led by First Nations, Métis, and Inuit people (#1)^c^, incorporate Indigenous world views and realities into research and methodology (#3)^c^, find creative solutions to problems (#7)^c^ | ✔ | ✔ | ✔ | ✔ | ✔ |  | ✔ | ✔ | ✔ | ✔ | ✔ | ✔ | ✔ |
| **Reducing bullying**  eg, workplace bullying and harassment (#1)^c^, bullying and violence in schools (#4)^c^, bullying as a risk factor (#11)^c^ bullying and cyberbullying prevention (#17)^c^ | ✔ | ✔ |  |  |  | ✔ | ✔ |  | ✔ |  |  | ✔ |  |

^a^MHA: mental health and addictions.

^b^LGBTQ2S+: Lesbian, Gay, Bisexual, Transgender, Queer or Questioning and Two-Spirit.

^c^The number following # in parentheses refers to the item number (policy document) in Multimedia Appendix 1.
