# Supplementary material for: Priority Given to Technology in Government-Based Mental Health and Addictions Vision and Strategy Documents: Systematic Policy Review
Source: J Med Internet Res. 2021 May 5;23(5):e25547. doi: 10.2196/25547 (PMC8135019; doi:10.2196/25547)
Supplement: Multimedia Appendix 3 [file jmir_v23i5e25547_app3.docx]

**Multimedia Appendix 3.** Frequency of technology-related categories mentioned by each province/territory.

| Number of categories mentioned by each province/territory out of the total 22 categories | Province/territory | | | | | | | | | | | | |
| --- | --- | --- | --- | --- | --- | --- | --- | --- | --- | --- | --- | --- | --- |
|  | Alberta | Newfoundland and Labrador | Yukon | British Columbia | Ontario | Saskatchewan | Nova Scotia | Nunavut | Manitoba | Prince Edward Island | Québec | New Brunswick | Northwest Territories |
| n | 14 | 14 | 11 | 10 | 10 | 10 | 9 | 5 | 3 | 3 | 3 | 2 | 1 |
